# “There doesn’t seem to be the help there for people who are carers”: qualitative findings from a realist evaluation of hospital-to-home transitions

**DOI:** 10.1093/geroni/igag056

**Published:** 2026-05-18

**Authors:** Lauren Lawson, Matthew Cooper, Clare Tolley, Annette Hand, Hamde Nazar

**Affiliations:** National Institute for Health and Care Research Newcastle Patient Safety Research Collaboration, Newcastle University, Newcastle upon Tyne, United Kingdom; National Institute for Health and Care Research Newcastle Patient Safety Research Collaboration, Newcastle University, Newcastle upon Tyne, United Kingdom; School of Psychology, Newcastle University, Newcastle upon Tyne, United Kingdom; School of Pharmacy, Newcastle University, Newcastle upon Tyne, United Kingdom; National Institute for Health and Care Research Newcastle Patient Safety Research Collaboration, Newcastle University, Newcastle upon Tyne, United Kingdom; School of Healthcare and Nursing Sciences, University of Northumbria, Newcastle upon Tyne, United Kingdom; National Institute for Health and Care Research Newcastle Patient Safety Research Collaboration, Newcastle University, Newcastle upon Tyne, United Kingdom; School of Pharmacy, Newcastle University, Newcastle upon Tyne, United Kingdom

**Keywords:** Unpaid caregivers, Dementia, Multiple long-term conditions, Hospital discharge

## Abstract

**Background and Objectives:**

Unpaid carers play a significant role in supporting older adults with multiple long-term conditions, including dementia (MLTCiD), during complex hospital-to-home transitions. However, their needs are often overlooked during hospital discharge, highlighting a gap in how meaningful support can be provided. This study explored how, for whom, and under which circumstances these transitions work (or not) for carers.

**Methods:**

Fifteen semi-structured interviews were conducted with carers as part of a realist evaluation. Interviews were coded to identify patterns of contexts and outcomes, explained through causal mechanisms, and used to refine an explanatory framework capturing what works, for whom, and under which circumstances in promoting safer transitions.

**Results:**

Three interdependent themes that shaped carer experiences were identified: Managing information, Carer well-being, and Engagement with support. Fragmented or inaccessible information increased carers’ uncertainty, reduced engagement, and negatively influenced well-being. Many carers felt overwhelmed by excessive or conflicting information and reported barriers to accessing reliable guidance, such as limited trustworthy sources and low digital literacy. Carers valued a single point of contact to coordinate information and provide relational support, but implementation of this role was rare.

**Discussion and Implications:**

Care during hospital-to-home transitions remains fragmented, with limited support for carers. Although digital solutions have the potential to improve efficiency and safety, current systems overlook carers’ roles, capacity, and needs. Involving carers in service design, improving communication, and providing personalized guidance could strengthen safety in transitional care.

Innovation and Translational Significance: This study uses a realist evaluation approach to understand how hospital-to-home transitions work for unpaid carers of older adults living with multiple long-term conditions, including dementia. It identifies 3 components that explain why carers feel unsupported by current discharge practices: information management, carer well-being, and engagement with support. This work is innovative in shifting focus from service processes to recognizing carers’ lived experiences and capacities. Findings can inform policy and practice by embedding carers in discharge planning, introducing a consistent point of contact, and guiding co-design of accessible, carer-centric information and digital tools to improve safety and continuity of care.

## Introduction

Dementia is characterized by progressive impairment that impacts everyday function, and is a leading contributor to disability and dependency among older adults (aged ≥65 years).^1^ In the United Kingdom (UK), approximately 1 in 3 people will care for someone living with dementia in their lifetime, contributing 1.34 billion hours of unpaid care each year.^2^ This equates to over £21.1 billion annually, representing the largest dementia-related cost and exceeding half of the total societal cost.^3^ Older adults with dementia often have additional long-term conditions, which contribute to frequent hospitalizations and repeated transitions between care settings.^4^^,^^5^ Many are discharged from the hospital, requiring greater care than at admission, increasing reliance on friends and family to provide support in the community as unpaid caregivers (referred to as carers).^6^^,^^7^

Hospital-to-home transitions are a vulnerable point in the care pathway and a known risk to patient safety, due to inadequate information transfer and communication breakdowns, increasing the risk of preventable readmission.^8^^,^^9^ Carers often facilitate continuity of care by coordinating information between providers and settings (i.e., location care is received).^8^^,^^10^ Their involvement can improve access to person-centered care and reduce readmissions, while insufficient support contributes to burnout and delayed patient recovery.^8^^,^^11^^,^^12^ Although UK legislation states that carers should be included in discharge planning, implementation is inconsistent, with evidence to suggest their involvement is rare.^6^^,^^11^^,^^13^^,^^14^ Current statutory guidance from the Department of Health and Social Care in the UK outlines expectations for practice, but does not address the specific circumstances enabling meaningful involvement.^8^ Understanding this inconsistency requires moving beyond identifying whether involvement occurs to explaining the specific contexts under which carer involvement can be achieved.

In a previous realist review, an explanatory framework identifying core components of successful hospital-to-home transitions for older adults with multiple long-term conditions, including dementia (MLTCiD), was developed.^15^ Findings from this review highlighted that fragmented, poorly coordinated transitions often shifted responsibility onto carers, largely without training or support, generating emotional strain, decision-making conflicts, and heightened risk of patient deterioration and subsequent readmission.^15^ However, the majority of documents included in this review explored statistical associations between MLTCiD and hospital readmission using administrative data. While these data are useful for developing a framework of core components, detail-rich qualitative data were necessary to further unpack which underlying, causal mechanisms are involved in this process, in order to test and refine this framework following a realist approach.^16^^,^^17^ Understanding carers’ experiences of navigating complex, poorly coordinated transitions is essential to determine what influences their capacity to provide support.^18^ By identifying contexts that enable or prevent involvement, and mechanisms that influence capacity to support patients, this study aimed to explain how, for whom, and under which circumstances hospital-to-home transitions work (or do not work) for carers of older adults with MLTCiD by understanding their perspectives of these transitions.

## Methods

### Design

Realist evaluation is a theory-driven approach that examines what works (or does not) in an intervention, for whom, under which circumstances, and why.^17^ Realist evaluations test program theories developed from existing literature, such as from realist reviews, that explain how interventions work and their expected impacts.^19^ Causality is inferred by identifying underlying causal mechanisms (M) linking specific contexts (C) to outcomes, forming and refining context-mechanism-outcome configurations (CMOCs) to understand how the intervention works.^19^ Realist approaches are suited to evaluating complex, context-dependent health and care programs, such as carers’ experiences of highly variable hospital-to-home transitions.^19^^,^^20^ This study followed RAMESES II reporting standards for realist evaluations (Supplementary Table 1).^21^

The overarching program theory from the previous realist review that the present study sought to refine assumes that successful hospital-to-home transitions rely on coordinated support involving carers.^15^ When recognized, informed, and included in discharge planning, carers can facilitate information flow, continuity, and safer patient recovery. Conversely, when excluded, unsupported, or underprepared, carers experience increased stress, unmet needs, and adverse events. Limited dementia training for healthcare professionals (HCPs) and inconsistent approaches to communicating dementia diagnoses undermined information transfer, effective discharge planning, and care continuity. While the program theory identifies processes underpinning successful transitions and recognizes carers’ roles, it does not explain how they experience and respond to these conditions in practice, or how this shapes transitional outcomes.

### Population, sampling, and recruitment

We included adult, unpaid carers supporting older adults with any self-confirmed dementia diagnosis, at least one other long-term condition, who had provided care during a hospital-to-home transition. Bereaved carers and those supporting individuals in care homes were excluded. Following a realist approach, sampling was purposive and theory-based, including participants with relevant lived experience.^15^^,^^22^ Guided by principles of information power, detail-rich qualitative data were collected to test specific assumptions about carers’ roles within the wider program theory through cross-case analysis.^23^ Recruitment aimed to maximize inclusivity and reach by engaging carers to participate through multiple routes, including local carers groups in the North East of England, voluntary sector networks, social media, and the Join Dementia Research register, between January and July 2025. Eight members of the patient and public involvement and engagement (PPIE) platform Valuing Our Intellectual Capital and Experience (VOICE) provided written feedback on a summary of the study concept, plans, and accessibility of participant materials via an opportunity posted on the VOICE platform (https://www.voice-global.org). Demographic data were not collected.

### Data collection

Qualitative, semi-structured interviews were conducted with carers via Microsoft Teams, telephone, or face-to-face, as part of a realist evaluation. A topic guide, informed by the wider program theory, explored carer involvement in hospital-to-home transitions, well-being support, the influence of MLTCiD, and the use of digital tools.^15^ Consistent with realist interviewing, the guide was iteratively refined to pursue emergent areas that could test the program theory.^24^ As adaptations were specific to individual experiences, there was no input from PPIE contributors. All participants provided written informed consent. Demographic data were self-reported, including carer age, sex, carer status, and care recipient characteristics (age, sex, dementia diagnosis, time since diagnosis, and number of long-term conditions). All interviews were recorded, transcribed verbatim, and anonymized.

### Data analysis

Analysis was iterative, allowing for exploration of emerging ideas to refine the program theory, consistent with a realist logic of analysis.^19^ Transcripts were uploaded into NVivo, a software tool to facilitate analysis. One researcher (LL) coded the data retroductively, combining deductive (informed by the program theory and existing CMOCs) and inductive (capturing new insights) coding.^25^ Codes were grouped into categories from which contexts, mechanisms, and outcomes were identified and organized into CMOCs. Existing CMOCs were considered for their relevance and potential for refinement when applied to the new data.^15^ All CMOCs were repeatedly refined as understanding developed and discussed within the research team (LL, MC, CT, AH, and HN) to assess relevance, and reach consensus on the final program theory.

### Ethical approval

This study was approved by the Faculty of Medical Sciences Research Ethics Committee, part of Newcastle University’s Research Ethics Committee (Ref: 2910/50284).

## Results

### Participants

Interviews were conducted with 15 carers between March and July 2025, representing a range of hospital-to-home transition experiences across different hospitals, patient support needs, and durations of care. Three interviews were held face-to-face, 4 via Microsoft Teams, and 6 via telephone, lasting an average of 41 minutes (range 24–69 minutes). Participants included 13 females and 2 males, aged between 27 and 83 years (M = 61.5, *SD* = 13.5). All were identified as White British. Most cared for a parent (*n* = 10), did not live with the care recipient (*n* = 11), and had provided care for 1.5–12 years. Participants reported spending approximately 20–34 hours (*n* = 4) or 50+ hours (*n* = 4) each week on caring responsibilities. Most participants reported caring for someone with Alzheimer’s disease (*n* = 6) or mixed Alzheimer’s disease and Vascular dementia (*n* = 6), with an average of 3.5 additional long-term conditions. Full participant characteristics are presented in Table 1.

**Table 1:** Participant characteristics.

Characteristic	Unpaid carer		Care recipient	
	*n* (%)	Mean (range)	*n* (%)	Mean (range)
Sex, female	13 (86.7)			
Age, years		61.5 (27-83)		
**Relationship to care recipient**				
Spouse/partner	3 (20)			
Child	10 (66.7)			
Other^a^	2 (13.3)			
**Living with a care recipient**	4 (26.7)			
**Time caring, years**		5 (1.5-12)		
**Hours per week**				
0-9 hours	3 (20)			
10-19 hours	3 (20)			
20-34 hours	4 (26.7)			
35-49 hours	1 (6.7)			
50+ hours	4 (26.7)			
Age, years				85.2 (76-96)
**Type of dementia**				
Alzheimer’s disease (AD)			6 (40.0)	
Vascular dementia (VaD)			1 (6.7)	
Frontotemporal dementia			1 (6.7)	
Mixed: AD + VaD			6 (40.0)	
Mixed (unknown)			1 (6.7)	
**Time since diagnosis, years (months)**				3.5 (0.6-8)
**Number of long-term conditions**				3.5 (1-6)

a Other denotes younger, familial relationships.

### Refined program theory

**Figure 1:**
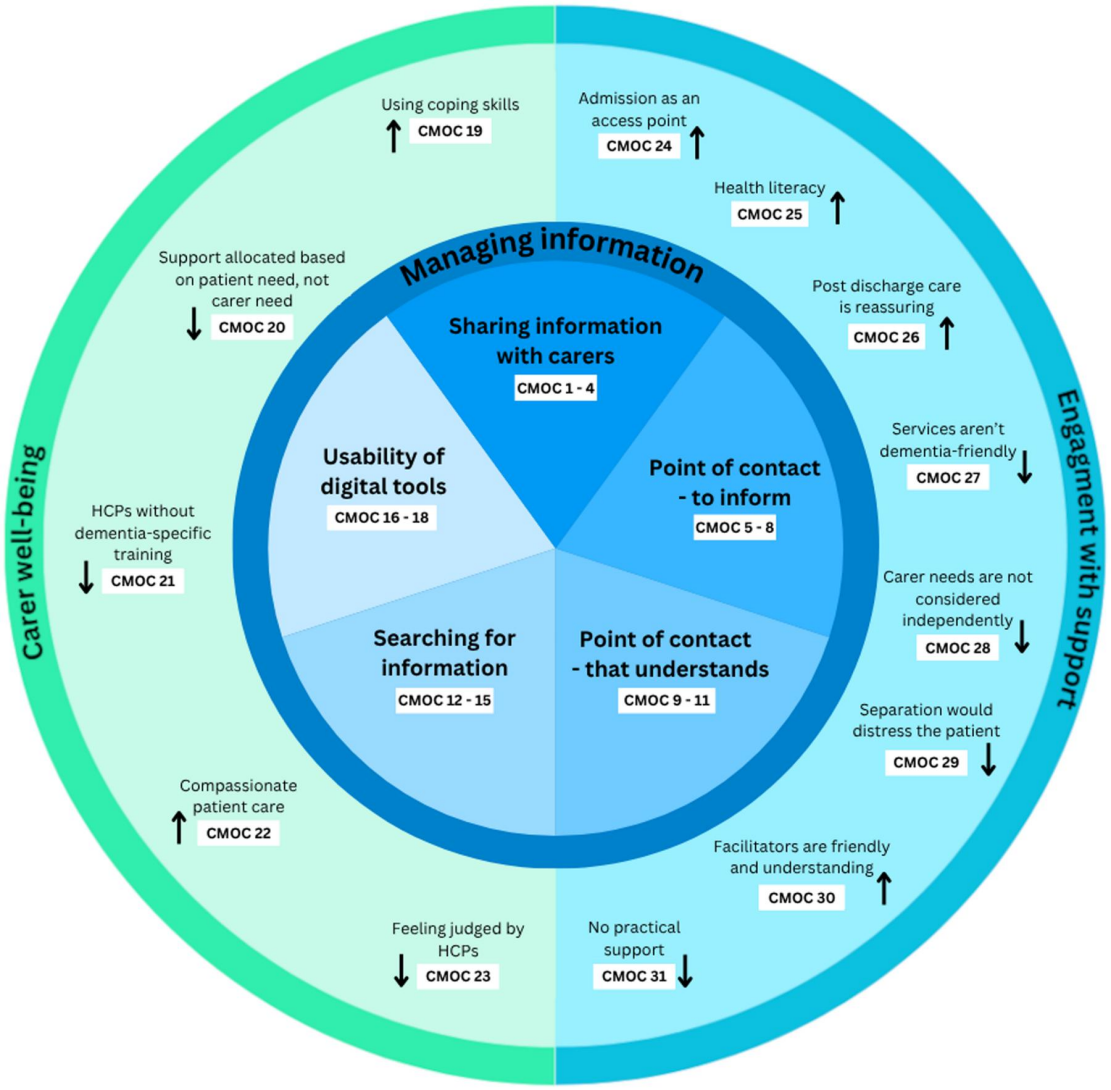
Refined program theory. Arrows denote positive or negative influence on well-being or engagement. CMOC = context-mechanism-outcome configuration.

Thirty-one CMOCs were developed under 3 broad themes: (*1*) *Managing information*; (2) *Carer well-being*; and (3) *Engagement with support. Managing information* was the central component influencing carers’ experiences of hospital-to-home transitions, and explains how health information about the patient was communicated to and from carers throughout the patient’s journey. This was further divided into 5 sub-themes: *1a) Sharing information with carers; 1b) Point of contact–to inform; 1c) Point of contact–to understand; 1d) Searching for information; and 1e) Usability of digital tools.* CMOCs developed from the interviews are available in Supplementary Material and have been explained below. The 3 themes were interdependent, where experiences of *Managing information* directly fed into *Carer well-being*, and carer *Engagement with support*. Figure 1 shows the refined program theory.

The refined program theory states:

Hospital-to-home transitions work well for carers when information about the patient’s health is clear, consistent, and coordinated because this supports carers to understand the patient’s needs, anticipate the care required at home, and feel involved in their care. When information is not shared, such as delayed/inconsistent updates from HCPs, carers experience uncertainty and distress. When a single point of contact can inform carers and provide relational support, they feel more confident and prepared to manage the transition. In the absence of a point of contact, carers search for information themselves, encountering conflicting guidance or digital systems that are difficult to navigate. This can trigger confusion, overwhelm, and reduced trust, undermining well-being and limiting engagement with support. Information management is central to carers’ experiences of hospital-to-home transitions, directly shaping whether those transitions can support carer well-being and engagement, or increase carer burden and isolation.

### Sharing information with carers

During the patient’s hospital stay, carers felt that HCPs did not routinely share information about the patient’s treatment with them. Despite frequently “*reminding people [HCPs] that we’re here because he’s got dementia*” Carer Participant (CP) 14, and “*it’s in the medical records*” CP12, carers noted an “*unawareness of a dementia diagnosis*” CP12. In their absence, carers were concerned that HCPs wrongly assumed patients were providing a handover of information about their care and subsequently would provide “*very little information*” CP11 to the carer directly. This led to carers feeling uninformed about the patient’s treatment and how to care for them at home (CMOC1):

If I’ve not been there when the doctors come round, and the doctors talk to her, and then they’ve gone away, and I have no idea what the doctor said. And I have no idea what I’m supposed to be doing with her. I can’t ask her because she doesn’t know, and she can’t remember. (CP5)

Carers described experiences of further fragmented information during transfer, where “*nobody [HCP] seemed to know what the forward treatment was*” CP5. As a result, carers felt stressed that their questions weren’t acknowledged (CMOC2). Most carers said they had not been involved in discharge planning and felt unsupported and unsure of how to care for the patient at home (CMOC3). Without discussing support needs with HCPs before discharge, carers felt unable to anticipate the help patients required, or what support they would need to facilitate this. “*When we got him home, he wasn’t mobile at all, really. And nobody had discussed any of that*” CP11. Some carers “*had no communication with the staff at all*” CP11 at the point of discharge. They described the process “*like a bus station or a transit lounge*” CP15, often stating, “*I felt very much just like patient transport.*” CP9. This resulted in poorer carer well-being and, in some cases, hospital readmission for patients (CMOC4):

I thought I could manage on my own without assistance, so no one came out after; we didn’t get any nursing care. […] But that was difficult, I found. […] I was burnt out, really. (CP7)

### Point of contact-to inform

Carers specifically wanted clear information about the patient’s condition, safety-netting advice, and signposting to the most appropriate service to contact if complications arose during transitions. This would improve their understanding of what to expect if the patient’s health deteriorated, and prepare them to respond (CMOC5):

What if he fell? What if he started to be unwell again? Do you go back to the hospital? Or do you go back to the general practitioner (GP)? […] if then a what if came up, yeah, you would know which way to go and who to contact. (CP1)

Carers who had received named contact details in hospital to provide them with information about the patient’s care suggested that extending this across the transition would improve their understanding of post-discharge care needs, as they would feel supported by knowing who to approach for help (CMOC6):

While she was actually in hospital, there was a named contact […] As soon as she left the hospital, he was no longer involved. So, while she was in hospital, you felt like it was all managed. But as soon as you left the hospital, you just kind of went into this black hole. (CP5)

When carers were contacted by multiple HCPs with different professional agendas who provided them with partial information about the patient’s care, they felt there was “*no coordination*” CP7, leading to them feeling overwhelmed (CMOC7). In addition, when HCPs provided inconsistent information about follow-up support, this led to carers feeling stressed and forgotten about (CMOC8).

### Point of contact–that understands

When carers were unsure of their role, they advocated for a point of contact that understood their experience, as “*having someone to talk to sometimes is all it takes.*” CP13. When this person could provide tailored information as well as emotional support, carers felt this would improve their understanding of how to care for the patient at home (CMOC10):

If I could just sit with somebody who understands and who could help me put something together […] answer some questions. Give me some more advice that I probably need. Just all of those things that you kind of touch on, but with lots of different people rather than one. (CP8)

Having a point of contact to act as a liaison between carers and HCPs was “*efficient and impactful*” CP2, providing reassurance that HCPs understood their perspective, which supported care decisions during discharge to be made in a timely manner (CMOC9). When they had a proactive general practitioner (GP) that could provide person-centered care, carers had increased trust that the GP could be relied upon, “*she became kind of like, you know, more invested, she would come, she would do home visits when other people wouldn’t*” CP9. This increased their likelihood of engaging with the GP for support if the patient’s health deteriorated (CMOC11). In contrast, carers without a supportive GP experienced difficulties navigating between condition specialists, reporting that “*you don’t get much interaction with them.*” CP5

### Searching for information

When carers needed more information about managing care, they reported searching online. However, finding information that was relevant to their local area was difficult, as not knowing what could be trusted increased the time spent searching across multiple sites (CMOC12):

I’ve always been on the Internet and found out what I could about dementia and how to deal with things and what you need to put in place, […] I found it helpful, but there’s a risk that you might be reading something that’s not been verified, and it’s a load of rubbish […]. So, you have to kind of look at several places and then take the amalgamation of everything. (CP5)

Carers reported frustration when online resources provided “*very general information. It’s not actually like, you know, it’s not to that specific person.*” CP13. As a result, they were less inclined to search beyond initial results as they felt the content of the information provided was not specific to their needs (CMOC13). They also noted how “*you haven’t got the time to read all this [general information]*” CP7 when caring for someone, and that this process added to their stress and ability to take in the most important information (CMOC14).

Carers also accessed information about available support during hospital-to-home transitions from community support groups. This was facilitated by groups that provided a welcoming space where carers could share their own experiences, as they trusted information from people “*who’ve been through this sort of situation themselves.*” CP11 (CMOC15).

### Usability of digital tools

Most carers were unaware of any digital tools that could support their needs during the hospital-to-home transition. They also often lacked confidence using digital technology, “*I’m sort of frightened to touch a button in case I mess it up.*” CP3, attributing this to being “*age related.*” CP1. This meant that they were less likely to use digital tools, “*there was some way of getting in touch with the doctor on the NHS or the NHS about looking at [patient]’s records, but I’ve never had to do that because it sounds so complicated to me.*” CP1 (CMOC16). Despite reservations, some carers were willing to adopt digital tools, “*I am a technophobe, but I’m willing to learn. But it has to be slowly.*” CP6, if support was available:

Who is there to help anyway? Get access to the NHS app? You know, obviously I do it for myself but […] if somebody could just kind of help like and sort it out for my mum, like, help me to do it. (CP4)

Carers described experiences where they were required to use digital platforms to access onward care and support, such as GP appointments or organizing disability benefits. However, structural barriers such as a lack of valid identification or poor internet connectivity meant that they would spend longer completing these tasks, which increased the burden and delayed their access to support (CMOC17):

I was actually trying to get my dad on the NHS app, but because he hasn’t got any photo ID, valid photo ID, I couldn’t set it up, and I’d have to go to his GP and sort it out. […] I don’t really use anything, anything online. (CP10)

Carers also explained how they had “*the NHS app myself on my phone, but I think you can’t have it for somebody else.*” CP4. Using the app was “*more difficult if you’re power of attorney because you don’t get the full access.*” CP5, which meant that carers were less likely to use the app when proxy access limited the information they could view (CMOC18):

I spoke to the GP today. And if I were on the NHS app, I could like check the results. But I can’t do any of that, so it’s all calls. (CP4)

### Carer well-being

“*As a carer, you’re constantly, your well-being is constantly pummeled.*” CP9. All carers acknowledged the negative impact caring had on their well-being, which was often exacerbated during transitions. Some reported using coping skills they had learned through support for their own health, which prepared them to manage stress when patients were discharged (CMOC19). However, when post-discharge support services were allocated based on the needs of the patient, carers who were themselves ageing, injured, or experiencing poor mental or physical health had limited opportunities for self-care. They were consequently more likely to neglect their own health, because they felt their responsibility for the patient was more important than their own well-being (CMOC20):

I was 2, 2 1/2 weeks out of having a hip replacement, and I had to take her out. I then had to care for her because there’s nobody else. I couldn’t get an agency. So, I put myself at risk of DVT or of a dislocated hip, etc., etc., because there is no option. (CP15)

In hospital, carers believed that HCPs lacked dementia-specific training, “*I think he was just sat in the chair and just left. And I don’t think they understood his needs at all*” CP11. This resulted in increased distress when interacting with health and care services, as carers felt helpless to ensure the patient’s needs were prioritized (CMOC21): “*I know that everybody’s busy and whatnot, but they just couldn’t. They said she wasn’t a priority.*” CP8. However, when HCPs could offer tailored, compassionate care for patients, carers felt understood and supported, which reduced their distress and enabled them to share responsibility for the patient’s care: “*They were there for us, so I felt totally safe and secure throughout the whole process*” CP6 (CMOC22). Carers also perceived judgment from HCPs when they disagreed with the care plan, which undermined their confidence in making decisions and raised concerns about whether this might affect future support if they were unable to provide care at home (CMOC23):

Rather than feeling supported. There’s probably a feeling of being judged, that’s what if I do? What if I’m not able to as my sister? What if my sister’s not able to cope? Or what if something doesn’t quite go according to plan? Then I will be judged for not being able to provide the care that they are saying should be provided. (CP2)

### Engagement with support

For carers who up until that point received no formal support, the patient’s hospital admission was “*a blessing in disguise*” CP10 that increased their awareness of available services, improving access to support (CMOC24). However, many carers reported having limited medical knowledge and felt that information during transitions was not adapted to their level of health literacy, “*they may treat me like a professional carer, but I’m not. They may treat me like a healthcare professional, but I’m not.*” CP15. They reported having to be proactive to access care for the patient, but reflected “*it’s hard to be proactive sometimes when you don’t know what you’re doing*” CP13. Carers with higher health literacy (e.g. healthcare workers) were confident advocating for patients, increasing their likelihood of accessing services and receiving support (CMOC25):

I knew off the back of my hand kind of straight away what to say. But when they’re asking like my mam the questions, she doesn’t work in healthcare, and she just like, didn’t really understand what they meant by what does she want at home? (CP12)

When post-discharge services could assist with activities carers felt unable to perform, such as moving and handling, they were more likely to engage with the support. This “*took a lot of stress away knowing that there were people coming in to help*” CP10, because carers felt reassured that post-discharge services knew how to handle patients, so shared responsibility (CMOC26). Carers described how community services intended for people with dementia were often not adapted for their needs, including lecture-style advice sessions, a lack of patient transport, and groups scheduled only in the morning. “*There’s one thing that you know […] it’s [morning] the worst time of the day*” CP9 (CMOC27).

Carers felt isolated when patients refused support (e.g., paid carers and community groups). This meant that they were less likely to engage with formal support for the patient or their own well-being, as their needs were not considered independently, “*he’s so difficult, you know […] It’s hard to sort of access any of those things.*” CP10 (CMOC28). Although carers were aware of respite care, they often felt that separation would cause the patient distress, so they were less likely to use this to support their own well-being (CMOC29). Carers who accessed these services benefitted from “*talk[ing] to another carer*” CP9. By fostering trust and belonging, these interactions promoted social connection. (CMOC30).

“*If you asked my dad, he would say there’s nowt[nothing] out there apart from a listening ear, which doesn’t do any good*” CP13. When carers already understood the patient’s needs, they spoke of frequent disappointment when social services could not offer practical support: “*Talking to somebody when nobody takes any action doesn’t get me anywhere*” CP15. Carers acknowledged that health and social care services were under pressure, but were reluctant to engage elsewhere after interacting with services that could not support their needs, as they felt little motivation that anything was available to help them (CMOC31):

There doesn’t seem to be any help there for people who are carers. (CP13)

## Discussion

### Summary of findings

This study explored how and under which circumstances hospital-to-home transitions work for carers of older adults with MLTCiD. Our program theory identified 3 interdependent components shaping carer experiences: *Managing information*, *Carer well-being*, and *Engagement with support*. Gaps in information exchange negatively influenced well-being and limited engagement, while tailored support and trusted communication could reduce uncertainty, promoting carer involvement. Findings indicate that how carers interpret and respond to contextual factors encountered during transitions determines whether transitions are successful.

### Comparison with existing literature

Information management was the central component influencing carer experiences. Fragmented, generic, or poorly timed information left carers underprepared and uncertain, negatively affecting well-being and leading to patient readmission in some cases. Similar findings have been reported elsewhere, with reduced access to appropriate materials, resources, and information for carers during hospital-to-home transitions linked to an increased risk of patients experiencing medication-related harm.^26^ Ensuring patients and carers receive the right information, in the right way, at the right time has been identified as a gap between policy and practice across the dementia care pathway.^27^ This finding reinforces the wider program theory assumption that poor information sharing across care settings is detrimental to successful transitions.^15^

Carers valued a single point of contact to coordinate generic information (e.g., post-discharge guidance and signposting), reduce uncertainty, and provide person-centered support. Although consistent with transitional care models, such roles were rarely implemented, as we reported previously in the wider program theory.^15^^,^^28^^,^^29^ This highlights an ongoing gap in service provision, where additional resources may be needed to develop a coordination role to support carers to receive information. Digital tools (e.g., NHS app) could support coordination of generic information, aligning with the shift to digital in the 10-Year Health Plan.^30^ However, few carers in this study used these tools, suggesting that a “digital first” approach may exacerbate digital exclusion, particularly among at-risk groups such as older carers with low confidence or poor access to technology.^30^^,^^31^ Although training could support this, a recent evaluation of the NHS app found that HCPs rarely received training to use it themselves, and were often unaware of available resources, limiting their ability to support carers.^32^ This demonstrates the need for approaches that are digital first but not digital only.

While proxy access via the NHS app can simplify medication management and improve access to patient health records,^32^ carers in this study described restricted access and difficulties coordinating onward care, which decreased engagement. The “My Carer” section proposed in the 10-Year Health Plan could address this by enabling timely advice and reassurance, though our findings suggest that strategies promoting active carer engagement are necessary to prevent further exclusion.^30^ Existing initiatives, such as digital hubs or collaboration between App Ambassadors and community organizations, may support inclusion.^30^ However, many carers in this study reported feeling isolated, and did not all engage with community groups, emphasizing the importance of involving them in the design of potential digital solutions, and the development of supportive strategies to facilitate engagement.^30^

Information given at discharge on the patient’s condition was often framed in clinical language, limiting understanding and access to support. Although statutory guidance requires HCPs to assess carers’ needs at hospital discharge, only 14% of those nationally surveyed reported being asked about their capacity to provide care.^33^ Further, the guidance does not specify how to deliver these discussions accessibly, reflective of different health literacy levels.^8^ Using plain language, minimizing jargon, and providing opportunities for clarification (e.g., checking back) enhances understanding among patients and carers at discharge, improving decision making.^26^^,^^34^^,^^35^ This supports our findings that health literacy is a contextual factor influencing carer engagement, and should be accounted for at hospital discharge. However, as most carers in this study reported having no contact with HCPs at discharge, there may be ambiguity in who, if anyone, holds responsibility for this role.

Hospital-to-home transitions increased stress, with many carers neglecting their own health and well-being to provide care. This is well-evidenced in the literature where caring has been identified as a social determinant of health, adversely affecting physical, emotional, and financial well-being.^33^^,^^36^ Other research has highlighted that carers’ well-being and opportunities for self-care must be considered alongside appropriate support and information during hospital-to-home transitions to facilitate more carer involvement and reduce burden.^26^ Although carers valued relational support and acknowledged pressures on health and care resources, they often viewed existing services as unfit for purpose, reducing motivation to engage further. From a realist perspective, interventions offering relational support will only function effectively under the correct structural context when responsibility for care coordination is clearly allocated and funded within health and care systems. Evidence of interventions to support carer well-being (e.g., respite, education, and support groups) remains limited, with methodological issues and inconsistent findings regarding effectiveness.^37^ More robust research is needed to develop effective interventions that deliver meaningful, sustainable support.

### Implications

Our findings emphasize a persistent failure to recognize carers’ needs as distinct from those of patients during hospital-to-home transitions. Formal integration of the carer role within patient care plans is necessary to ensure systematic support for carers is embedded within discharge pathways. Carers should receive timely, accessible, and relevant information to reduce uncertainty and enable effective care. Digital tools could function as a touchpoint for centralized information management, but robust and effective implementation must include support for those with limited digital literacy, alongside non-digital alternatives to mitigate the risk of exclusion.^30^ Carers rarely prioritized their own well-being and frequently perceived available services as inadequate. Although the 10-Year Health Plan’s shift of care into the community may improve continuity, this transition risks increasing carer burden without appropriate resources to provide meaningful, practical support, with evidence to suggest 44% of carers were concerned this shift would increase their responsibilities.^30^^,^^33^ As carers are already less likely to prioritize their own well-being, this may increase the number of carers living with poorer health as an unintended consequence, who may be less likely to seek support.^38^ Overall, carer engagement was shaped by both information management and well-being, highlighting the need for transitional care interventions to integrate tailored, sustainable support as a core component of successful discharge planning.^15^

### Strengths and limitations

This study contributes evidence to a novel program theory explaining the contextual factors involved in successful hospital-to-home transitions for older adults with MLTCiD. Consistent with realist methodology, we sought to include participants with varied experiences to identify relevant causal mechanisms.^22^ Despite multiple recruitment strategies, most participants were White British, female carers from areas of low-to-medium deprivation. Recruiting carers is a recognized challenge in dementia research.^39^ Recruitment of carers from deprived areas or minoritized ethnic backgrounds was challenging, emphasizing well-documented barriers to research participation.^40^ While our sample broadly reflects UK census data on carers, this limits transferability to other social groups where mechanisms may operate differently.^41^ Further testing with more diverse carers would strengthen theory refinement. Although realist approaches account for complexity, findings represent a partial understanding of hospital-to-home transitions, based on available knowledge.^17^ This study forms part of a multi-method realist evaluation designed to refine this understanding and build upon a wider program theory. By explaining how carers influence the success of hospital-to-home transitions and the contextual factors shaping this process, this study supports the development of guidance to promote successful transitions.

## Conclusion

Managing information plays a central role in shaping carers’ experiences of hospital-to-home transitions when supporting older adults with MLTCiD. Fragmented, inaccessible, and poorly timed information was detrimental to carers’ well-being and limited their engagement with support. Digital tools and community-based support offer potential solutions to this complex problem, but risk widening existing inequalities if implemented without understanding carers’ diverse needs. Future research should prioritize methods of ensuring carer involvement throughout hospital-to-home transitions whilst supporting their well-being.

## Supplementary Material

igag056_Supplementary_Data

## Data Availability

This study was not preregistered. Due to the risk of identifying participants, additional data are not available.
