# Supplementary material for: “There doesn’t seem to be the help there for people who are carers”: qualitative findings from a realist evaluation of hospital-to-home transitions
Source: Innov Aging. 2026 May 18;10(8):igag056. doi: 10.1093/geroni/igag056 (PMC13344844; doi:10.1093/geroni/igag056)
Supplement: igag056_Supplementary_Data [file igag056_supplementary_data.pdf]

Innovation in Aging **Supplementary Material: Lawson et al. "There doesn't seem to be the help there for people who are carers": Qualitative findings from a realist evaluation of hospital-to-home transitions.**

Supplementary Table 1. List of items to be included when reporting realist evaluations.

**TITLE**			**Page(s) in document**
1		In the title, identify the document as a realist evaluation	1
SUMMARY OR ABSTRACT			
2		Journal articles will usually require an abstract, while reports and other forms of publication will usually benefit from a short summary. The abstract or summary should include brief details on: the policy, programme or initiative under evaluation; programme setting; purpose of the evaluation; evaluation question(s) and/or objective(s); evaluation strategy; data collection, documentation and analysis methods; key findings and conclusions<br>Where journals require it and the nature of the study is appropriate, brief details of respondents to the evaluation and recruitment and sampling processes may also be included<br>Sufficient detail should be provided to identify that a realist approach was used and that realist programme theory was developed and/or refined	2
INTRODUCTION			
3	Rationale for evaluation	Explain the purpose of the evaluation and the implications for its focus and design	4
4	Programme theory	Describe the initial programme theory (or theories) that underpin the programme, policy or initiative	4, 5
5	Evaluation questions, objectives and focus	State the evaluation question(s) and specify the objectives for the evaluation. Describe whether and how the programme theory was used to define the scope and focus of the evaluation	4, 5
6	Ethical approval	State whether the realist evaluation required and has gained ethical approval from the relevant authorities, providing details as appropriate. If ethical approval was deemed unnecessary, explain why	7
METHODS			
7	Rationale for using realist evaluation	Explain why a realist evaluation approach was chosen and (if relevant) adapted	6
8	Environment surrounding the evaluation	Describe the environment in which the evaluation took place	6
9	Describe the programme policy, initiative, or product evaluated	Provide relevant details on the programme, policy or initiative evaluated	6
10	Describe and justify the evaluation design	A description and justification of the evaluation design (i.e. the account of what was planned, done and why) should be included, at least in summary form or as an appendix, in the document which presents the main findings. If this is not done, the omission should be justified and a reference or link to the evaluation design given. It may also be useful to publish or make freely available (e.g. online on a website) any original	6

		evaluation design document or protocol, where they exist	
11	Data collection methods	Describe and justify the data collection methods – which ones were used, why and how they fed into developing, supporting, refuting or refining programme theory<br>Provide details of the steps taken to enhance the trustworthiness of data collection and documentation	7
12	Recruitment process and sampling strategy	Describe how respondents to the evaluation were recruited or engaged and how the sample contributed to the development, support, refutation or refinement of programme theory	6, 7
13	Data analysis	Describe in detail how data were analysed. This section should include information on the constructs that were identified, the process of analysis, how the programme theory was further developed, supported, refuted and refined, and (where relevant) how analysis changed as the evaluation unfolded	7
RESULTS			
14	Details of participants	Report (if applicable) who took part in the evaluation, the details of the data they provided and how the data was used to develop, support, refute or refine programme theory	8
15	Main findings	Present the key findings, linking them to contexts, mechanisms and outcome configurations. Show how they were used to further develop, test or refine the programme theory	8 - 16
DISCUSSION			
16	Summary of findings	Summarise the main findings with attention to the evaluation questions, purpose of the evaluation, programme theory and intended audience	16
17	Strengths, limitations and future directions	Discuss both the strengths of the evaluation and its limitations. These should include (but need not be limited to): (1) consideration of all the steps in the evaluation processes; and (2) comment on the adequacy, trustworthiness, and value of the explanatory insights which emerged.<br>In many evaluations, there will be an expectation to provide guidance on future directions for the programme, policy or initiative, its implementation and/or design. The particular implications arising from the realist nature of the findings should be reflected in these discussions	18, 19
18	Comparison with existing literature	Where appropriate, compare and contrast the evaluation's findings with the existing literature on similar programmes, policies or initiatives	16 - 18
19	Conclusion and recommendations	List the main conclusions that are justified by the analyses of the data. If appropriate, offer recommendations consistent with a realist approach	19
20	Funding and conflict of interest	State the funding source (if any) for the evaluation, the role played by the funder (if any) and any conflicts of interests of the evaluators	20

Supplementary Table 2. Table of Context-Mechanism-Outcome Configurations

Number	Context-Mechanism-Outcome Configuration (CMOC)
**Managing information: 1a) Sharing information with carers**	
CMOC1	IF HCPs rely on the patient to provide accurate information when the carer has left the hospital, and do not share updates with the carer (C), THEN carers feel concerned that the patient is not receiving appropriate care for their illness (O), BECAUSE carers feel HCPs don't listen to them when they inform them about the patient's dementia (M).
CMOC2	IF carers don't have access to HCPs with up-to-date knowledge of the patient's treatment (C), THEN carers feel stressed about the patient's care pathway (O), BECAUSE they feel like their questions aren't being acknowledged (M).
CMOC3	IF carers do not receive information about the patient's plans for discharge from HCPs (C), THEN carers have no opportunity to reduce their feeling of confusion & uncertainty (O), BECAUSE they feel unsupported when care has been handed over to them (M).
CMOC4	IF carers don't get the opportunity to have an informed discussion about the patient's care needs at discharge (C), THEN they do not receive support in the community (O), BECAUSE the carer's reality is not considered (M).
**Managing information: 1b) Point of contact – to inform**	
CMOC5	IF carers receive clear information and signposting about what might happen to the patients' health after discharge, and who to contact at which point (C), THEN they have a better understanding about what to expect and how to support the patient at home (O), BECAUSE they feel more informed about what to do if the patient's condition deteriorates (M).
CMOC6	IF carers are provided with named/department contact details from the hospital after discharge (C), THEN carers have an improved understanding of the patient's post-discharge care needs (O), BECAUSE carers feel supported by knowing who can help them (M).
CMOC7	IF carers receive contact from multiple services without advanced notice, that do not provide clear information on how they will be supporting the patient (C), THEN they will be less likely to engage with information (O), BECAUSE they feel overwhelmed (M).
CMOC8	IF carers do not receive timely, consistent follow-up care from HCPs (C), THEN they experience increased stress (O), BECAUSE carers feel forgotten by the HCPs whilst waiting for the next steps in the patient care (M).
**Managing information: 1c) Point of contact – to understand**	
CMOC9	IF there is a point of contact responsible for coordinating post-discharge care to act as a liaison between carers and HCP teams (C), THEN care decisions can be made in a timely manner (reducing carer stress) (O), BECAUSE carers feel reassured that HCPs have a greater understanding of their situation (M).
CMOC10	IF a point of contact can provide carers with guidance and emotional support that is relevant to their experience (C), THEN carers will understand how to care for the patient (O), BECAUSE they feel heard when a point of contact can empathise with their experience (M).
CMOC11	IF individual GP practices provide person-centred care that is outside of their regular responsibilities (C), THEN carers will be more likely to engage with the GP for support if the patient's health gets worse (O), BECAUSE having a proactive GP that understands the patient's needs increases trust that carers can rely on them (M).
**Managing information: 1d) Searching for information**	
CMOC12	IF carers don't think they have enough information to manage care after hospital discharge, so use online sources of information (C), THEN they may spend more time searching and combining information from multiple sites (O), BECAUSE they don't know what to trust (M).
CMOC13	IF carers believe that available resources provide general information that is not specific to their needs (C), THEN they won't use online resources when they need support (O), BECAUSE they feel frustrated (M).
CMOC14	IF the information carers receive after discharge is inaccessible and it takes time to find what is relevant to them (C), THEN they are less likely to engage with information (O), BECAUSE they feel overwhelmed (M).

Number	Context-Mechanism-Outcome Configuration (CMOC)
CMOC15	IF support groups provide a welcoming space for carers to share the knowledge they have about supporting someone after hospital discharge (C), THEN carers have increased opportunities to learn about what support is available (O), BECAUSE carers trust information from people with shared experiences (M).
**Managing information: 1e) Usability of digital tools**	
CMOC16	IF (older) carers do not have experience or knowledge of using digital health technologies (C), THEN they will rely on non-digital methods (e.g. phoning GP, in-person visits) (O), BECAUSE they lack confidence in their ability to use unfamiliar technology (M).
CMOC17	IF carers are required to use digital platforms (e.g. NHS app, government website) to access support services, but face structural barriers (poor internet connection, no valid ID for patient) (C), THEN carers experience increased burden, and it takes them longer to access support (O), BECAUSE using the digital platforms places higher demand on their time (M).
CMOC18	IF carers do not have full access to the patient's data on the NHS app (e.g. test results, repeat prescriptions, discharge summaries) (C), THEN carers will be less likely to engage with the app spend more time contacting HCPs to get the information they need (O), BECAUSE they feel the app can't give them the information they want (M).
**2) Carer well-being**	
CMOC19	IF carers have been taught coping skills from support for their own health (C), THEN they are better able to cope in stressful caring situations (O), BECAUSE they feel better prepared to cope when supporting someone with dementia (M).
CMOC20	IF carers are ageing, injured or experiencing poor mental or physical health, but support services (e.g. respite care) are allocated based on the needs of the patient (C), THEN carers have limited opportunities for self-care, and are more likely to neglect their own health (O), BECAUSE they feel responsible for the patient, prioritising their needs ahead of their own (M).
CMOC21	IF carers believe HCPs lack dementia-specific training and are too busy to provide compassionate care (C), THEN carers feel increased distress engaging with healthcare services (O), BECAUSE carers feel helpless to ensure the needs of the patient are prioritised (M).
CMOC22	IF HCPs in hospital offer compassionate care tailored to people with dementia (C), THEN carers feel less distress as the patient is less distressed (O), BECAUSE carers feel understood, and can share responsibility with the HCPs (M).
CMOC23	IF carers do not agree with care plans put forward by HCPs in hospital (C), THEN carers feel less sure they are making the 'right' choice and will receive support if something changes (O), BECAUSE they feel HCPs are negatively judging their ability to provide care (M).
**3) Engagement with support**	
CMOC24	IF carers receive no support when caring for the patient prior to the hospital admission (C), THEN the admission can serve as a turning point for additional support to be organised (O), BECAUSE the carers' awareness of what support is available is increased (M).
CMOC25	IF carers have medical knowledge about the patient's care needs (C), THEN they are more likely to access services and receive support (O), BECAUSE they feel confident advocating for the patient (M).
CMOC26	IF post-discharge care is organised to assist with home-based activities that carers are unable to do themselves (C), THEN their stress is reduced (O), BECAUSE they feel reassured that the paid carers know what they're doing, so let them take charge (M).
CMOC27	IF community support is not adapted to the needs of the patient (C), THEN carers will not engage with the services (O), BECAUSE they don't think the services understand their needs (M).
CMOC28	IF the patient refuses support services (e.g., paid carers, community groups) that require their consent to proceed (C), THEN the carer is less likely to access respite or support (increasing emotional distress) (O), BECAUSE the needs of the carer are not considered independently of those of the patient (M).

Number	Context-Mechanism-Outcome Configuration (CMOC)
CMOC29	IF carers feel like they cannot be separated from the patient (C), THEN they are less able to access respite to improve their own well-being (O), BECAUSE carers believe taking a break would be distressing for the patient (M).
CMOC30	IF carers interact with support services (e.g., community groups, paid carers, counselling) where facilitators can demonstrate friendliness, empathy, and understanding through shared experience (C), THEN they are more likely to engage with the services (O), BECAUSE carers feel socially connected and supported emotionally through building trust and belonging (M).
CMOC31	IF carers reach out to services for support, but don't receive any practical help that addresses their needs (C), THEN they will be less likely to engage with services, increasing isolation (O), BECAUSE carers feel less motivated to engage when nothing supports their needs (M).
